# A Challenging Case of Closing Gastroschisis With Gangrenous Bowel: A Case Report

**DOI:** 10.7759/cureus.91515

**Published:** 2025-09-03

**Authors:** Aqeela J Madan, Shaikha Janahi, Fayza Haider

**Affiliations:** 1 Pediatric Surgery, Salmaniya Medical Complex, Ministry of Health, Manama, BHR; 2 Pediatric Surgery, College of Medicine and Medical Sciences, Arabian Gulf University, Manama, BHR

**Keywords:** abdominal wall defect, case report, closing, congenital, gastroschisis, neonate

## Abstract

Gastroschisis is a rare congenital anomaly characterized by herniation of abdominal viscera through a right-sided abdominal wall defect without a protective membrane, typically identified during prenatal imaging. One of its most serious forms, known as closing gastroschisis, may result in bowel strangulation and significant clinical challenges. We report the case of a Yemeni male neonate delivered at 34 weeks of gestation via emergency cesarean section following premature rupture of membranes. At birth, the infant presented with a small abdominal wall defect and herniated bowel that appeared dusky, gangrenous, and foul-smelling. Emergency laparotomy revealed extensive bowel necrosis requiring resection and stoma formation. Although the postoperative period was initially stable, the neonate developed cholestasis associated with total parenteral nutrition (TPN) and a stoma prolapse, both of which contributed to feeding intolerance. Despite supportive management, the infant died at three months of age due to intestinal failure-associated liver disease. This case illustrates the complexity of managing closing gastroschisis and underscores the importance of early intervention, careful nutritional planning, and long-term supportive strategies to improve outcomes.

## Introduction

Gastroschisis (GS) is a rare congenital malformation [1] and the most common type of congenital abdominal wall defect [2]. The term *gastroschisis* in the ancient Greek language is known as *cleft belly* [3]. GS is defined as “a congenital malformation characterized by visceral herniation usually through a right-side abdominal wall defect to an intact umbilical cord not covered by a membrane” [3], with a typical defect size of 2-4 cm [2]. The pathogenesis of GS is not completely understood, although it is assumed to be caused by a disruption in the migration of the lateral ventral body folds early in embryonic development, resulting in a para-midline deficit [4], as one of the two omphalomesenteric arteries that connect the yolk sac to the dorsal aorta recedes [1]. The worldwide incidence of GS is reported to be 1 in every 3268 births (3 to 4.5 per 10,000 live births) with marked regional variation [5]. GS is usually diagnosed antenatally during the first trimester (12-14 weeks of gestation) or the second trimester (18-21 weeks of gestation) anomaly scans, by detecting free-floating bowel (without membrane) outside the abdominal cavity and an abdominal wall defect distinct from the umbilical cord insertion location [6], during these anomaly scan the size and the type of the defect can be determined [7]. According to studies, GS is more common with young maternal age [8], young mothers who smoke and consume alcohol during pregnancy [9]. Omphalocele, where the intra-abdominal organs are herniated inside the umbilical cord, resulting in a deformity of the midline abdominal wall [2], is a common differential of GS once the patient with an abdominal wall defect is born. In cases of simple GS, which involve no gastrointestinal complications, the survival rate following surgical intervention is approximately 90% [5]. However, in complex or closed GS cases, which occur in about 6% of all GS cases [10], the abdominal wall defect becomes partially or completely closed around the herniated bowel in utero, leading to bowel ischemia due to compromised blood flow [7]. This severe form is associated with a significantly higher mortality rate, reaching up to 70% [10]. GS is managed neonatally either by primary closure or delayed secondary closure [11]. We report a challenging and unique case of a closing GS with successful initial management and surgical intervention despite a unique presentation of gangrenous and twisted bowel pedicle.

## Case presentation

We present a Yemeni male who was delivered via emergency lower segment cesarean section (EMLSCS) at 34 weeks of gestation due to premature rupture of membranes. The Apgar scores were 9, 10, and 10 at 1, 5, and 10 minutes, respectively. His birth weight was 2.530 kg. He was antenatally diagnosed with an abdominal wall defect and labeled as GS. He was born to a 24-year-old woman, gravida 3, para 1, with one previous abortion.

**Figure 1:**
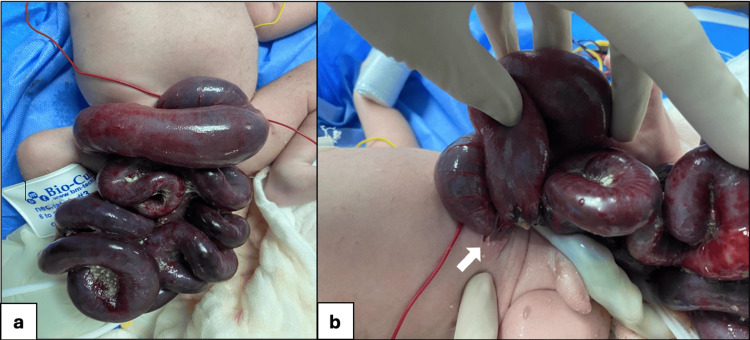
Preoperative findings of eviscerated bowel through a small abdominal wall defect. (a) Eviscerated small and large bowel appearing dusky, edematous, and gangrenous, with a blue-black discoloration, covered with debris and fibrinous material. (b) Preoperative image showing a small abdominal wall defect (<2.5 cm) to the right of the umbilicus, with an intact umbilical stalk and fusion between the abdominal wall skin and the serosa of the prolapsed bowel. The white arrow indicates the location of the defect.

**Figure 2:**
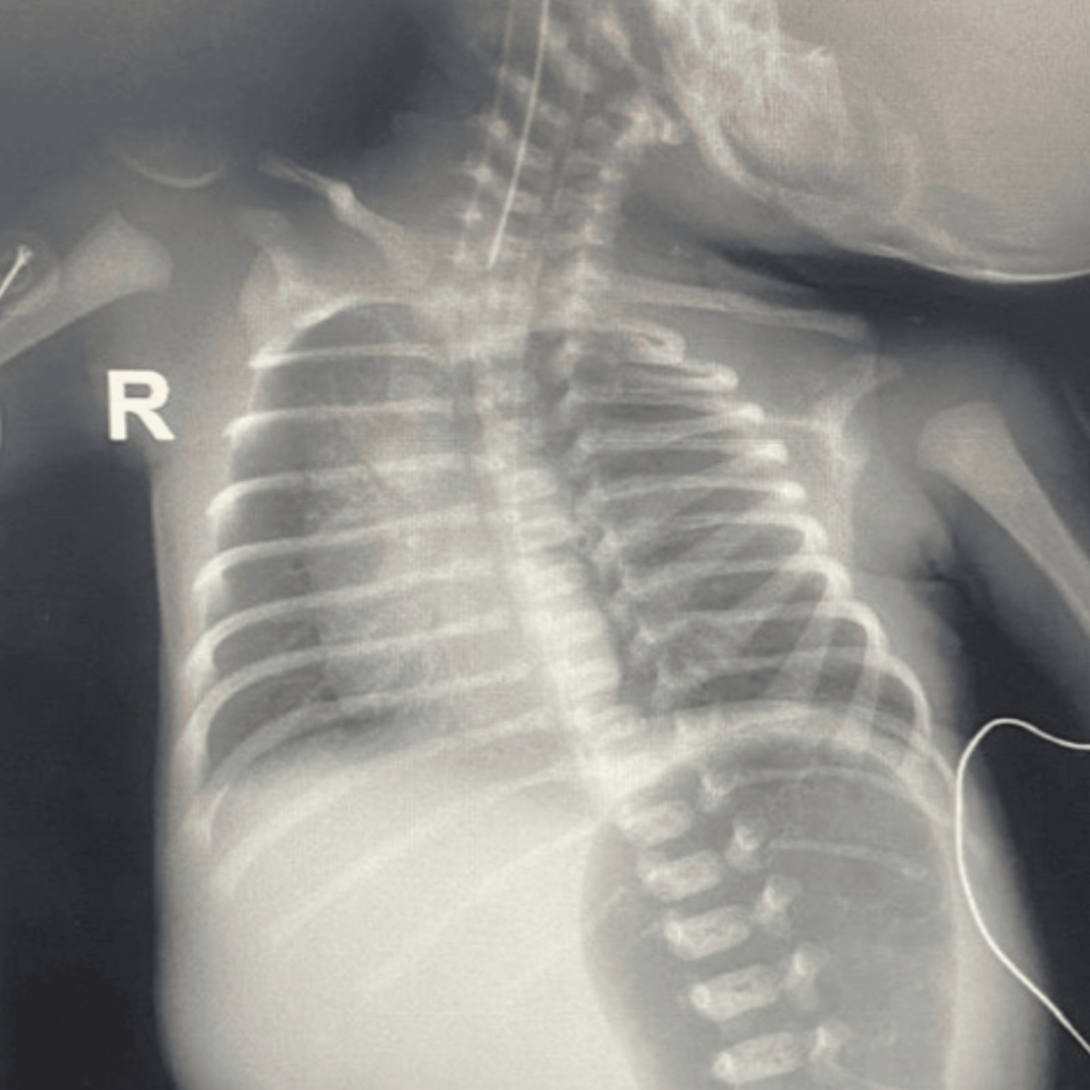
Preoperative abdominal X-ray demonstrating herniation of bowel loops through the umbilical defect. X-ray showing herniation of small and large bowel loops through a small umbilical abdominal wall defect. Air is seen in the dilated stomach. No solid organ herniation is evident.

Immediately after birth, the bowel was noted to be dusky, gangrenous, and foul-smelling (Figure 1a). On physical examination, he had normal male external genitalia. There was evisceration of both small and large bowel loops, which appeared edematous, bluish to blackish in color, and covered with debris and fibrinous material. The abdominal wall defect was located to the right of the umbilicus, measured less than 2.5 cm, and lacked an identifiable sac. There was fusion between the abdominal wall skin and the serosa of the prolapsed bowel, suggesting a diagnosis of closing GS (Figure 1b). The external genitalia were normal male. An abdominal X-ray (Figure 2) confirmed herniation of small and large bowel loops through the abdominal wall defect without other associated congenital anomalies. The patient was intubated on low ventilator settings, sedated, and managed with intravenous fluids (IVF). The exposed bowel was covered with warm, sterile, saline-soaked gauze, and an orogastric tube (OGT) was inserted for bowel decompression. The neonate was subsequently reviewed and cleared for surgery by the anesthesia team.

**Figure 3:**
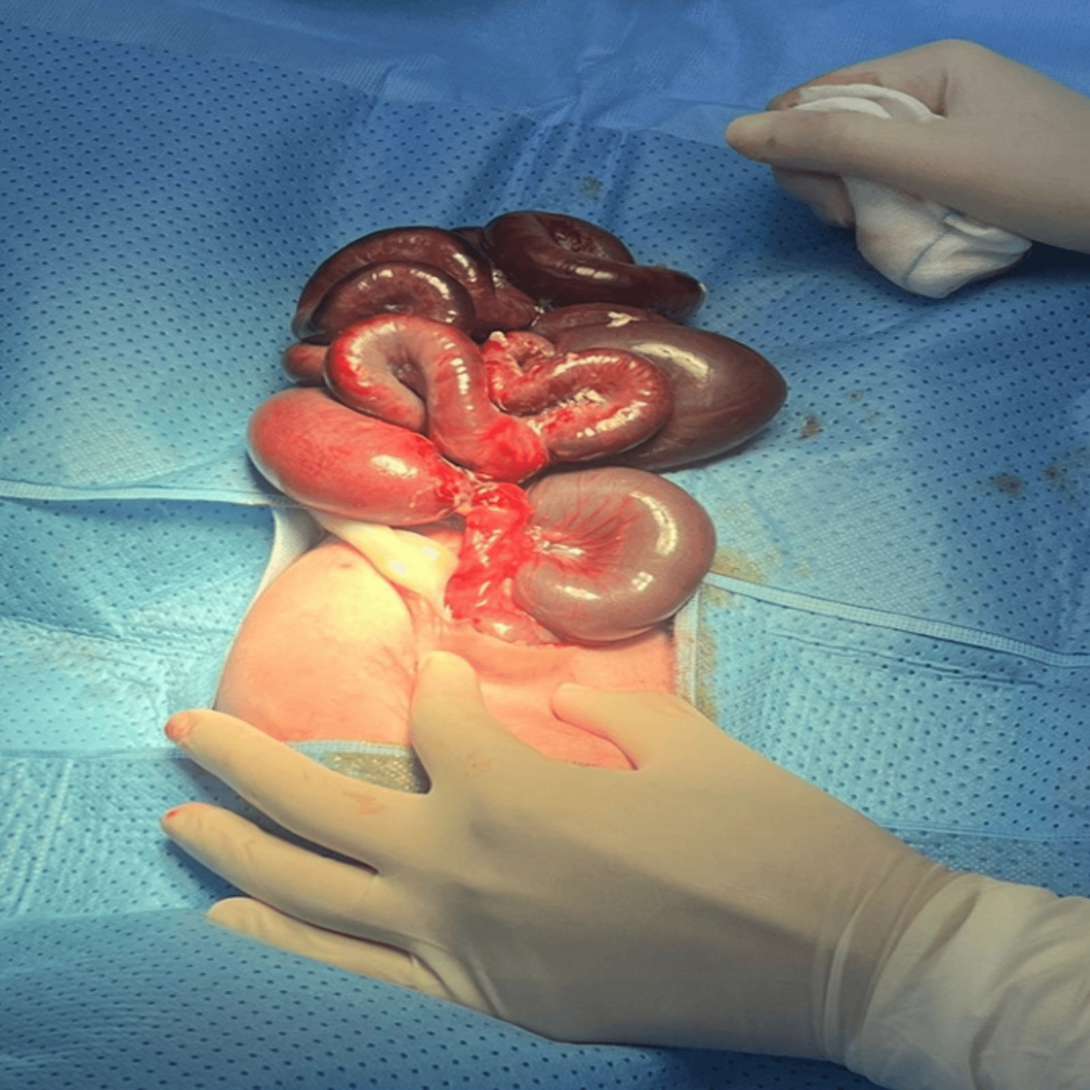
Intraoperative findings of bowel gangrene with volvulus and dilated bowel segments. Intraoperative image showing edematous, dusky, and partially gangrenous bowel with fibrinous exudate. The small bowel and abnormal large bowel (cecum, appendix, and transverse colon) are dilated, with a twisted pedicle between the gangrenous and healthy segments.

**Figure 4:**
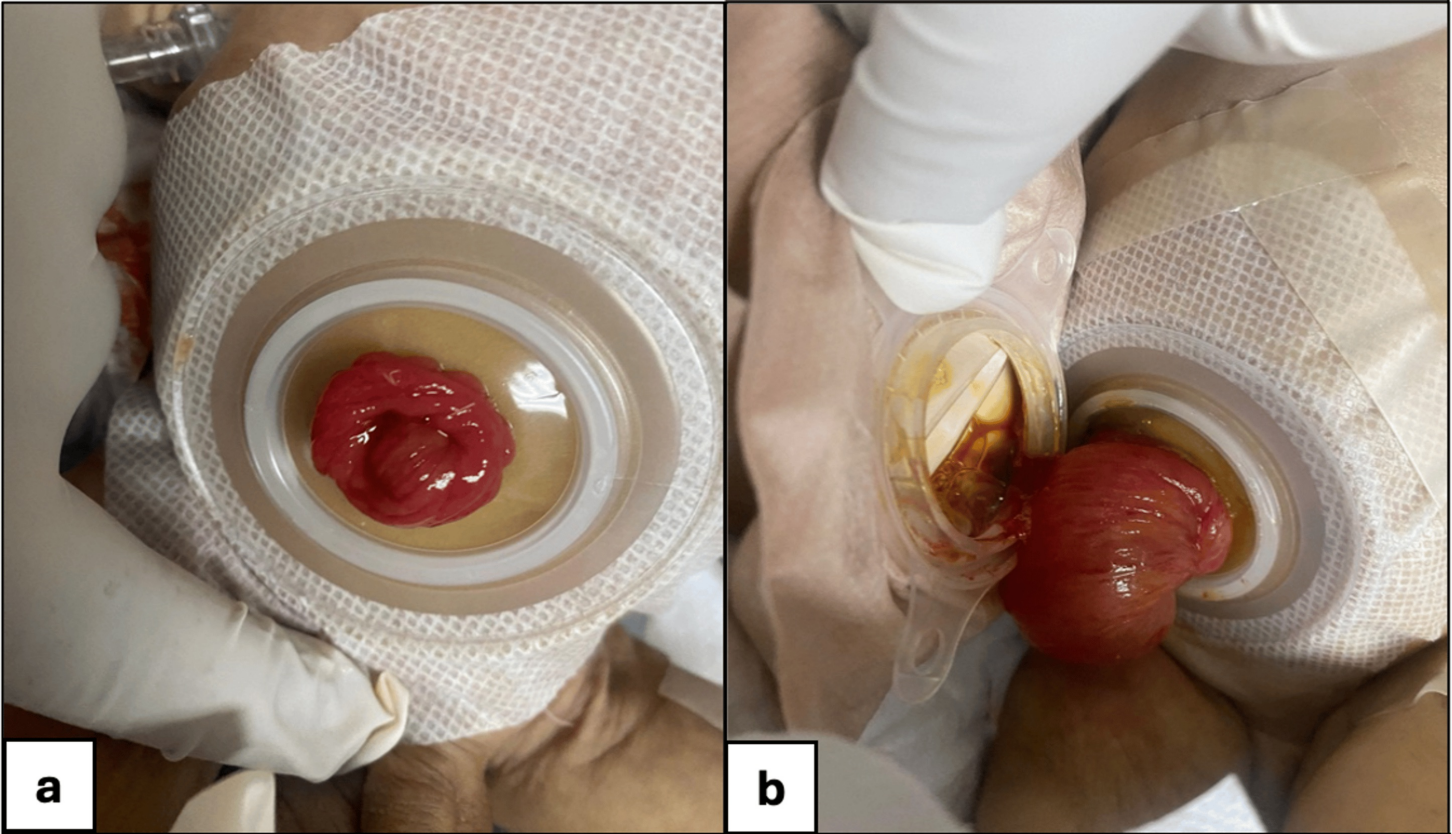
Postoperative ileostomy outcomes: normal vs. prolapsed stoma presentation. (a) Postoperative clean ileostomy with dressing intact, without prolapse or oozing. (b) Prolapsed stoma measuring approximately 7 cm at 30 days postoperatively

The patient was taken immediately to the operating theater within one hour after counselling and consenting. Intraoperatively, we found a large amount of herniated small and large bowel through a very small abdominal wall defect, less than 2.5 cm, at the right side of the umbilicus, with fusion between the abdominal wall skin and serosa of the prolapsed bowel (Figure 1b). The bowel was edematous, dusky, and black in some areas (gangrenous), covered with fibrous material, and matted (Figure 3). The umbilical stalk was intact (Figure 1b), containing two arteries and one vein. Under aseptic conditions, with the patient fully prepped and draped, a right transverse para-umbilical incision was made. Approximately 90 cm of dilated, gangrenous small bowel and abnormal large bowel (including the cecum, appendix, and transverse colon) were resected, with a twisted pedicle between the gangrenous segment and healthy bowel (Figure 3). The stomach was massively distended, atonic, and filled with coffee-ground fluid, aspirated by anesthesia through OGT. Approximately 40 cm of small bowel remained from the duodenojejunal junction (DJ) and 10 cm of large bowel. No free fluid or perforation was observed. The bowel was evacuated of meconium, and the bladder was visualized and deflated. No other abdominal organ anomalies were noted. After closure of the GS, an ileostomy was created and a stoma bag was placed (Figure 4a).

**Figure 5:**
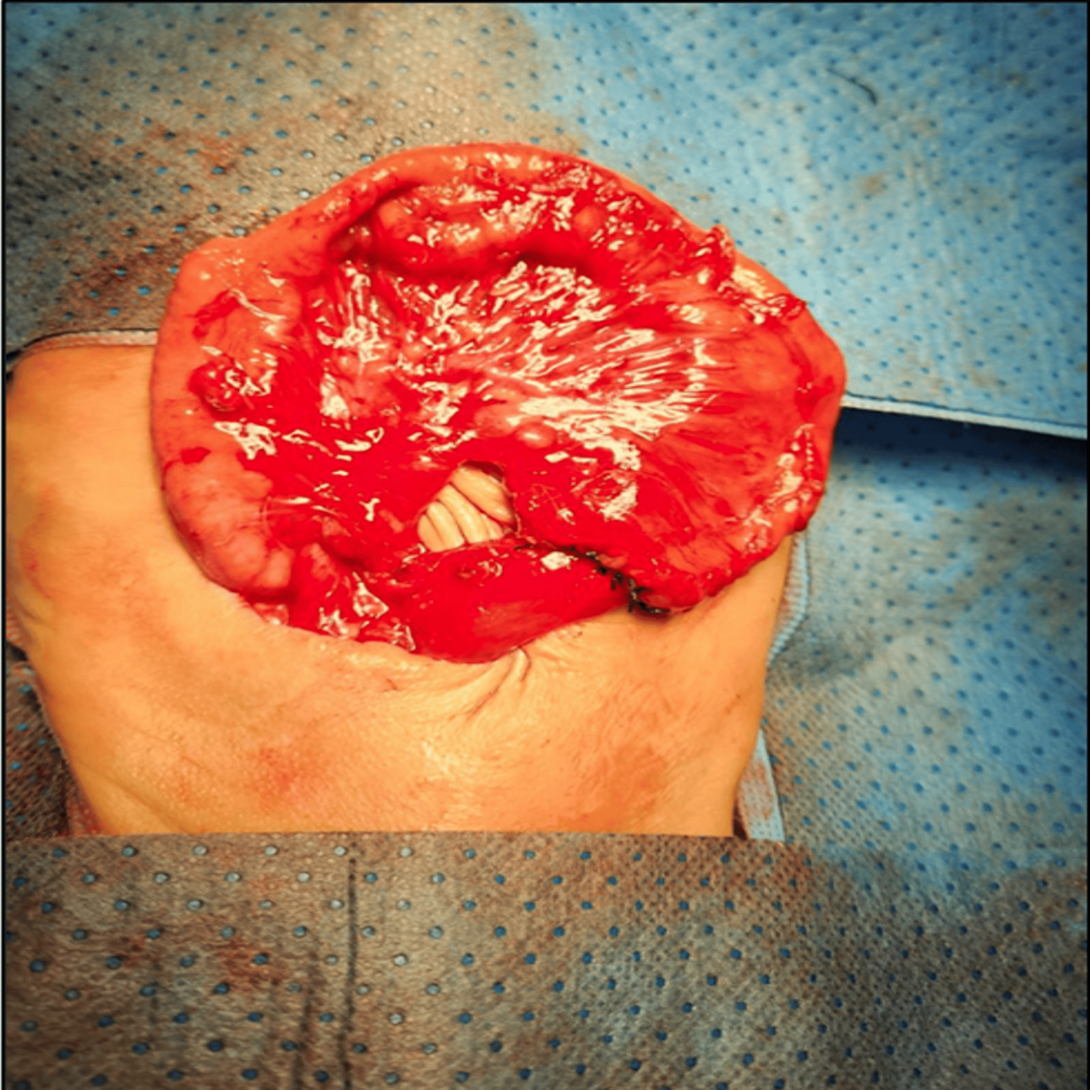
Post-resection intraoperative view showing healthy bowel and completed anastomosis after stoma closure. Remaining healthy bowel following resection and anastomosis (stoma closure performed after six to seven weeks). The size discrepancy between the small bowel (4 cm) and large bowel (1.5 cm) was managed with an end-to-end anastomosis.

The post-op patient was admitted to the neonatal intensive care unit (NICU) for further monitoring. Initially, he was doing well with no complications. The patient was on a peripherally inserted central catheter (PICC) line, and total parenteral nutrition (TPN) was started. Stoma started to function on day 5 post-op. Soon after the feed was started, the patient developed TPN-associated cholestasis. His stoma started to prolapse at day 30 post-op, showing around 7 cm of prolapsed bowel (Figure 4b). The stoma was closed because of a high-output stoma and large prolapsed bowel at two months of age. The discrepancy of the small and large bowel (4 cm vs. 1.5 cm) was managed appropriately by end-to-end anastomosis (Figure 5). Post closure of the stoma, he continued to have bowel dilatation with OGT showing greenish aspirate; the bowel of the neonate was not opened. However, we could not restart feeds as he developed feed intolerance, and the patient suddenly arrested at three months of age. We hypothesize that he developed intestinal failure-associated liver disease (IFALD) due to TPN-associated cholestasis.

## Discussion

Managing GS is extremely challenging. A rapid and multidisciplinary approach is needed to achieve greater outcomes. GS is a heterogeneous congenital abdominal wall defect [12], characterized by a 2- to 4-cm abdominal defect through which the internal organs herniate [3], typically on the right side of the umbilicus, without a covering membrane [13]. Translating the word gastroschisis literally would be the separation of the stomach, but in reality, it is not the stomach that separates, but likely the amniotic-ectodermal connection or an amniotic rupture at the right edge of the umbilical ring [14]. GS is believed to arise from early embryonic disruption [15]. In one study published in 2008, they hypothesized that the primary defect in GS results from a failure of the yolk sac and associated vitelline structures to integrate into the body stalk [16]. Despite all suggested hypotheses, the exact etiology of GS is unknown. The incidence is increasing, reaching approximately 3 to 4.5 per 10,000 live births [5]. 

Closing GS is a rare complication of GS where the abdominal wall closes over the herniated bowel, causing strangulation, with a high mortality rate (10%-70%) [14]; therefore, a rapid and multidisciplinary team approach is needed. It is believed to be caused by exposure to digestive compounds in amniotic fluid or mesenteric constriction, resulting in complications like infarction, ischemia, atresia, stricture, or resorption, which may lead to short bowel syndrome (SBS) [17]. Closing GS can lead to various outcomes, ranging from a closed abdominal ring with viable viscera to severe complications like type III intestinal atresia, midgut infarction, resorption, or a normal-appearing abdominal wall, known as vanishing midgut [17].

Risk factors of GS include young maternal age, as documented by most of the published case reports [9]. Genetic studies indicate that only 1.2% of GS cases involve chromosomal abnormalities [12], suggesting that environmental factors play a significant role in its pathogenesis. Maternal smoking, as well as the use of substances like marijuana, cocaine, methamphetamine, and certain depression medications, has been linked to an increased incidence of GS [12]. Environmental pollutants and pesticide exposure are also considered high-risk factors [9]. Additionally, infections such as herpes simplex virus and maternal psychological stress may contribute to the development of this congenital defect [12]. 

Diagnosis of GS is confirmed by antenatal ultrasound as early as 12 weeks of gestation, as it can readily detect GS in a fetus, with the bowel floating in the amniotic fluid and lacking a covering membrane [12]. Although diagnosis often occurs before delivery, the prognosis during the prenatal period is generally favorable, with a low incidence of intrauterine fetal demise [8].

Initial management of GS is crucial, and the main goal is to limit umbilical vein access to preserve physiological stability, provide respiratory support, maintain body temperature, and protect the bowel [12]. As reported in our case, the exposed bowel should be covered with sterile saline-soaked gauze, and a nasogastric tube should be placed for intestinal decompression. To compensate for fluid loss, a 10% glucose sodium chloride solution may be administered, while monitoring vital signs, capillary refill, and urine output to ensure proper organ perfusion [12].

Several surgical techniques have been documented in the literature, and these include primary closure, staged closure, and plastic sutureless closure [18]. The primary facial repair or closure under general anesthesia is the oldest surgical technique [12]. Silo-based reduction is considered the second evolution after primary closure, with less need for ventilatory support and general anesthesia. It reduces post-op NICU stay and enhances recovery and is recommended for GS with a large abdominal defect and a large amount of eviscerated bowel [12]. In recent years, the bedside reduction technique using sutureless closure has become increasingly prevalent. This approach involves intentionally leaving the umbilical cord long, utilizing it as a biological dressing to cover the defect following the reduction of abdominal contents. The sutureless repair shares the theoretical benefit of staged silo reduction, as the fascia remains open during the early postnatal period [12]. This could not be applied to our case as the eviscerated bowel was gangrenous.

While as many as 20% of individuals with GS may be unable to undergo primary fascial closure because the defect is too large and there is excessive herniation of intra-abdominal organs [7], only one study [10], consisting of two cases and excluding our study, has reported use of the primary closure technique, as the defect and amount of herniated bowel were small.

In our case, we proceeded with primary closure because most of the herniated bowel was gangrenous and required resection, which left us with sufficient intra-abdominal space for reduction and anastomosis creation. Our technique demonstrated successful initial outcomes and good feed tolerance. An important step is rapid management; in our case, the patient was operated on within one hour after delivery and confirmation of GS, with the parents actively involved in the surgical decision-making.

In the literature, there are six case reports of closed GS and two case series. The first case series, published in 2008 [17], was a retrospective study that included six patients with closing GS. Only one patient died at 9 months of age from parenteral nutrition-associated cholestatic liver disease, similar to our case. The second case series, also published in 2008 [17], included 17 patients who underwent the sutureless procedure for closing GS and 28 who underwent primary closure. This study demonstrated different surgical approaches and their impact on patient outcomes and follow-up, concluding that sutureless closure is effective for the diverse presentations of GS, with a success rate of 95%. It was associated with reduced duration of mechanical ventilation and general anesthesia use, as well as a shorter NICU stay.

Six case reports have described neonates with closing GS, highlighting the diverse presentations of this condition. Bowel atresia and obstruction were common findings [10,14,15], with complex cases such as small bowel atresia and colonic atresia requiring resection and anastomosis. Most cases required early surgical intervention within hours of birth, including silo placement, bowel reduction, resection, and anastomosis [10,18,19]. Closure of GS was achieved through techniques such as silo bag placement with progressive reduction [19] or delayed closure with reinforcement [18]. In cases involving bowel atresia [10,12], surgical management included resection of dilated bowel segments with anastomosis. More complex cases required additional procedures such as stoma formation [14,15], as in our case. Postoperative complications were also reported, with several cases developing infections, including sepsis and Candida infections [14,15,18]. In addition, total parenteral nutrition (TPN)-related complications such as cholestasis and liver failure due to prolonged TPN were notable in some reports [10,15], consistent with our case. Necrotizing enterocolitis and stoma complications were also common, as we reported stoma prolapse in our case. In some cases, the infants showed good recovery with normal development [18,19]. These cases share common themes of early surgical intervention, the need for postoperative care, and the management of complications such as infections, TPN-related issues, and bowel function.

As seen in our case, the primary closure of the GS was successful, as the amount of remaining bowel was sufficient to close the abdominal wall without fear of abdominal compartment syndrome. Moreover, the patient initially tolerated feeds without any complications or signs of obstruction or intolerance. Eventually, the baby developed IFALD due to TPN-associated cholestasis, as reported in other case reports [10,15].

Our case is notable for presenting a rare congenital anomaly rarely seen in live-born infants. Unlike other cases, this child experienced strangulation of the extruded intestines, leading to gangrenous, dusky, and foul-smelling bowel that required resection and anastomosis due to a narrowed abdominal wall defect. Another notable aspect is the unexpected postoperative survival, given the limited intestinal surface available for absorption. One limitation we faced while managing this case was the sudden deterioration of the infant. He was initially tolerating feeds but eventually developed IFALD due to TPN-associated cholestasis. Further studies must focus on the proper adjustment of parenteral nutrition and feed tolerance post-GS repair to avoid serious complications.

## Conclusions

This case underscores the complexities and challenges involved in managing GS in neonates, particularly when compounded by complications such as bowel strangulation. It highlights the critical importance of timely surgical intervention, vigilant monitoring for potential complications, and the role of coordinated multidisciplinary care in improving outcomes. Nutritional support following surgical repair also emerged as a key aspect of management. Despite these insights, the case is limited by the absence of genetic testing, which restricts the ability to rule out underlying syndromic or chromosomal conditions, and by limited follow-up investigations before the patient’s clinical deterioration, which hinders a more comprehensive understanding of the condition’s progression and complications. These limitations should be taken into account when interpreting the findings and drawing broader conclusions.
